# Antidepressant-Like Effects of Cordycepin in a Mice Model of Chronic Unpredictable Mild Stress

**DOI:** 10.1155/2014/438506

**Published:** 2014-12-23

**Authors:** Zhang Tianzhu, Yang Shihai, Du Juan

**Affiliations:** ^1^Changchun University of Chinese Medicine, Changhcun 130117, China; ^2^Jilin Agricultural University, Changchun 130118, China; ^3^School of Life Science, Peking University, Beijing 100871, China

## Abstract

Cordycepin (3′-deoxyadenosine), a major bioactive component isolated from *Cordyceps militaris*, has multiple pharmacological activities. This study is attempted to investigate whether cordycepin (COR) possesses beneficial effects on chronic unpredictable mild stress- (CUMS-) induced behavioral deficits (depression-like behaviors) and explore the possible mechanisms. ICR mice were subjected to chronic unpredictable mild stress for 42 consecutive days. Then, COR and fluoxetine (FLU, positive control drug) were administered for 21 consecutive days at the last three weeks of CUMS procedure. The classical behavioral tests, open field test (OFT), sucrose preference test (SPT), tail suspension test (TST), and forced swimming test (FST), were applied to evaluate the antidepressant effects of COR. Then the serotonin (5-HT) and noradrenaline (NE) concentrations in hippocampal were evaluated by HPLC; tumor necrosis factor-*α* (TNF-*α*) and interleukin-6 (IL-6) in hippocampal were evaluated, and the proteins of TNF-*α*, IL-6, NF-*κ*BP65 5-HT_2A_ receptor (5-HT_2A_R), and brain-derived neurotrophic factor (BDNF) in hippocampal were evaluated by Western blot. Our results indicated that 6 weeks of CUMS exposure induced significant depression-like behavior, with low 5-HT and NE levels, high TNF-*α* and IL-6 in brain and high hippocampal TNF-*α*, IL-6, P-NF-*κ*BP65, and 5-HT_2A_R levels, and low BDNF expression levels. Whereas, chronic COR (20, 40 mg/kg) treatments reversed the behavioral deficiency induced by CUMS exposure, treatment with COR normalized the change of TNF-*α*, IL-6, 5-HT, and NE levels, which demonstrated that COR could partially restore CUMS-induced 5-HT receptor impairments and inflammation. Besides, hippocampal BDNF expressions were also upregulated after COR treatments. In conclusion, COR remarkably improved depression-like behavior in CUMS mice and its antidepressant activity is mediated, at least in part, by the upregulating BDNF and downregulating 5-HT_2A_R levels and inflammation in hippocampus.

## 1. Introduction

Depression is one of the most common mental disorders characterized by feelings of sadness, and major depressive disorder is a primary cause of disability [1]. It is the fourth major cause of morbidity worldwide at present and it will become the second by 2020 according to the World Health Organization [2]. Although there are many clinical effective antidepressant medications, most of them generate severe side effects. Therefore, it is necessary to develop new antidepressant drugs with lower adverse effects and better efficacy.

Plenty of clinical reports suggest that prolonged exposure to life stressful episodes, as a common risk factor, could provoke the development of major depression [3–5]. Currently, scientists adopt chronic unpredictable mild stress (CUMS) procedure which is performed such that animals are consecutively exposed to a series of unpredictable mild stressors to simulate a series of life stress events [6]. Indeed, a large number of ethological symptoms and neurobiological abnormalities found in CUMS-induced animals are similar to those exhibited in human depressed patients [7]. Thus, CUMS-induced depressive animal model has good validity and reliable predictability to screen new antidepressants through a series of behavioral tests.

The 5-HT_2_ receptors in the CNS are thought to be involved in psychiatric disorders such as depression, anxiety, sleep disorders, and hallucinations [8, 9]. Increased densities of cortical 5-HT_2A_ receptors are observed upon postmortem examination of depressed patients [10].

An increasing body of evidence presented in recent years has revealed that activation of the inflammatory response system plays a role in the pathophysiology of depression [11, 12]. Several studies reported increased levels of proinflammatory cytokines, for example, tumor necrosis factor-alpha (TNF-*α*) and interleukin-6 (IL-6) in depressive disorders [11, 12].

Cordycepin (3′-deoxyadenosine), a major bioactive component isolated from* Cordyceps militaris*, has multiple pharmacological activities, such as anti-inflammatory and immunomodulatory effects. Cordycepin prevents lipopolysaccharide- (LPS-) induced airway neutrophilia in mice and effectively blocks LPS-induced expression of vascular adhesion molecule-1 (VCAM-1) in the human epithelial cell line A549 [13]. Cordycepin inhibits interleukin-1*β*- (IL-1*β*-) induced matrix metalloproteinase-1 (MMP-1) and MMP-3 expressions in rheumatoid arthritis synovial fibroblasts (RASFs) and significantly inhibits AP-1 activation [14]. While there is little evidence regarding the relevance of COR on CUMS-induced depression, in this study we hypothesized that COR would improve CUMS-induced depression through regulation of r5-HT_2A_ receptor and BDNF.

## 2. Materials and Methods

### 2.1. Animals

Male ICR mice weighing 18–22 g (same batch) were purchased from Experimental Animal Center in Jilin University (Changchun, China, SCXK-0003). Prior to any experimentation, the mice were allowed to have one week to acclimatize to the laboratories. And, during the whole study, the mice were housed in group in constant laboratory conditions at a temperature of 22°C and 60% relative humidity under a 12 h light/12 h dark cycle. In our study, all the experimental procedures and laboratory animal care were performed in accordance with the National Institutes of Health (NIH) Guide.

### 2.2. Drug and Treatment

COR was purchased from Nanjing Jiancheng Co., Ltd. Fluoxetine hydrochloride (positive control drug) was obtained from Jinlin drugstore (Changhun, China). COR and fluoxetine were dissolved in 0.03% sodium carboxymethyl cellulose (CMC-Na). Mice were randomly divided into five different groups: one control group; one CUMS-vehicle (0.03% CMC-Na) group; one CUMS-FLU (15 mg/kg) group; and three CUMS-COR (20, 40 mg/kg) groups; the doses selection of COR was according to Zhang et al.'s report [15]. Each group included 20 mice. Every morning, all drug treatment groups were administered orally via intragastric gavage in a dose of 10 mL/kg body weight.

### 2.3. CUMS Procedure

**Figure 1:**
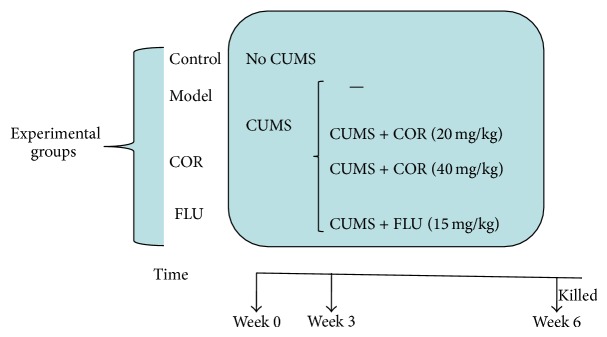
The protocol in this study.

The CUMS procedure was performed as described by Willner et al. [16], with a modification. Briefly, mice in the CUMS groups were exposed to different stressors, namely, food deprivation, water deprivation, empty water bottles (after water deprivation), cage tilt, grouped housing, soiled cage, stroboscopic lighting, restricted access to food (only give a small amount of food after food deprivation), 5 min cold swimming (at 15°C), 1 min tail pinch (1 cm from the end of the tail), physical restraint, illumination, and white noise. One of these stressors (in random order) was given every day for 6 weeks. The control group mice were left undisturbed except for necessary procedures such as routine cage cleaning and the protocol of the study can be seen in Figure 1.

### 2.4. Sucrose Preference Test (SPT)

Sucrose preference test was carried out at the end of week 0, week 3, and week 6, respectively. The method was performed as previous report [17]. In brief, 72 h before the test, a mouse was individually placed into cage with two bottles of sucrose solution to adapt to sucrose solution (1%, w/v) for 24 h; then one bottle of sucrose solution was replaced with water (24 h); after the adaptation, laboratory mice were deprived of water and food (24 h). Sucrose preference test was conducted at 9:30 a.m. Each mouse was housed in individual cage and was free to access two bottles containing 100 mL of sucrose solution (1% w/v) and 100 mL of water, respectively. After 12 h, the consumed weights of sucrose solution and water were recorded. The sucrose preference value was obtained from the following formula: sucrose preference (%) = sucrose intake (g)/[sucrose intake (g) + water intake (g)] × 100%.

### 2.5. Open Field Test (OFT)

The open field test was used to evaluate the locomotor activity of mice (crossing: horizontal movement scores reflect range of motion; rearing: vertical movement scores reflect exploratory behaviors). Mice were placed individually in the middle of an open field apparatus in a wooden box (40 × 60 × 50 cm) with the floor of the arena divided into 12 equal squares [18]. Following 2 min acclimatized to the apparatus, the numbers of crossings and rearings were counted in a 3 min session. After each trial, the open field apparatus was cleaned.

### 2.6. Tail Suspension Test (TST)

At the end of week 0, week 3, and week 6, respectively, the tail suspension test was performed based on the previous method that the mouse was hung 15 cm above the floor by the tip of the tail (1 cm) and was adhered to the lever [19]. The total test procedure of mouse immobility time was counted during a test period of 6 min (prior 2 min to adapt and recorded the last 4 min). And, only when the mouse was in a passively suspended and completely motionless status, it could be regarded as immobile.

### 2.7. Forced Swimming Test (FST)

Forced swim test was carried out at the end of the 6-week CUMS procedure. The test session was similar to that described in a previous report [20]. Briefly, each mouse was placed individually in an open cylindrical vitreous tank (height 20 cm, diameter 14 cm) containing 15 cm depth of water at 25 ± 2°C. All mice were forced to swim for 6 min, and the total duration of immobility was recorded during the last 4 min of the test. The definition of immobile status was that the mouse was floating in the water without any movement; only small motions are required to maintain its head above the water.

### 2.8. Measurement of 5-HT and DA Levels in the Hippocampus

Twenty-four hours after completion of the final food consumption test, six of the mice in each group were used for tissue assays of 5-HT and DA. The animals were decapitated and their brains rapidly were removed and dissected on an ice-chilled glass plate to obtain the hippocampus according to Franklin and Herberg [21] and Paxinos et al. [22]. Each tissue sample was weighed and homogenized by sonication in 200 *μ*L 0.4 M perchloric acid. The homogenate was kept on ice for 1 h and then centrifuged at 12,000 rpm (4°C) for 20 min. The supernatant was preserved, and the concentrations of 5-HT and DA were measured using high performance liquid chromatography with electrochemical detection (HPLC-ECD) as described by Qi et al. [23] with minor modifications. The mobile phase consisted of 85 mM citrate, 100 mM sodium acetate, 0.9 mM octyl-sodium sulfate, 0.2 mM EDTA, and 12% methanol, pH 3.7. An LC-10A pump (Shimadzu, Kyoto, Japan) was operated at 1.0 mL/min. The detector (L-ECD-6A, Shimadzu) was set at *þ*0.60 V. External standard curves were used to quantify the amounts of 5-HT and DA in each sample, calculated using the area under the curve. The injection volume was 20 *μ*L.

### 2.9. Measurement of TNF-*α* and IL-6 Levels in the Hippocampus

IL-6 and TNF-*α* levels in the hippocampus were measured by ELISA kits (DRG international, USA) according to the manufacturer's instructions.

### 2.10. Western Blot Analysis

Mouse hippocampus was chopped into small pieces and homogenized in ice-cold RIPA buffer containing 0.1% phenylmethylsulfonyl fluoride. The dissolved proteins were collected from the supernatant after centrifugation at 12000 g for 20 min. Protein concentrations were determined using Coomassie blue based assay reagent. Protein extracts were separated by a SDS-polyacrylamide gel electrophoresis and then transferred onto a PVDF membrane. The membrane was blocked with 5% skim milk in tris buffer saline and then incubated at 4°C overnight with respective primary antibodies for anti-5-HT_2A_R antibody (1 : 1000), anti-BDNF antibody (1 : 1000), anti-P-NF-*κ*BP65 (1 : 1000), anti-NF-*κ*BP65 (1 : 1000), anti-TNF-*α* (1 : 1000), anti-IL-6 (1 : 1000), and GAPDH (inner control, 1 : 1000). After washing with tris buffered saline, tween 20 (TBST), the membranes were incubated with a horseradish peroxidase conjugated secondary antibody (1 : 12000) for 1.5 h at room temperature. The antibody-reactive bands were visualized by using enhanced chemiluminescence detection reagents and a gel imaging system (Tanon Science & Technology Co., Ltd., China).

### 2.11. Statistical Analysis

All data were normally distributed and are presented as mean ± S.E.M. In the case of single mean comparison, data were analyzed by a Student's *t-*test. In the case of multiple mean comparisons, the data were analyzed by ANOVA and the Newman-Keuls post-test or two-way repeated measures ANOVA, followed by Bonferroni multiple comparison tests. *P* values less than 0.05 were regarded to reflect a significant difference.

## 3. Results

### 3.1. Effects of COR on the Percentages of Sucrose Consumption

**Figure 2:**
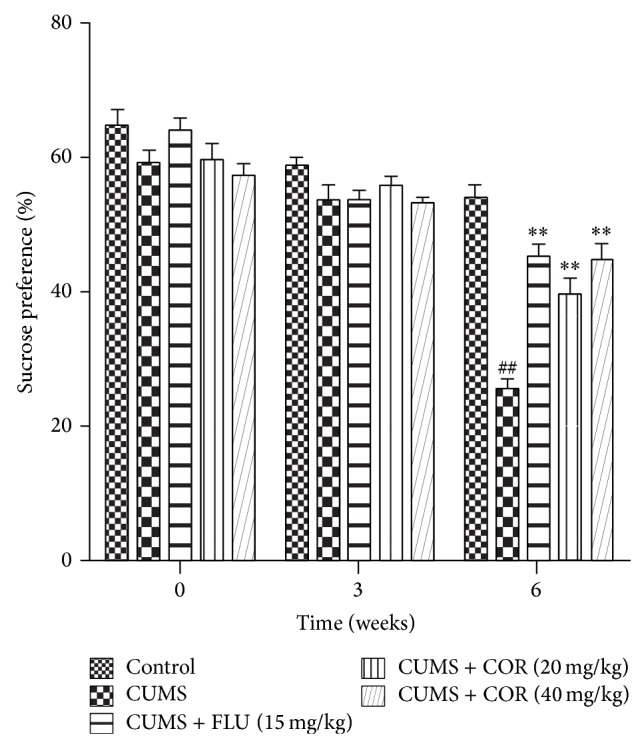
Effect of chronic COR administration on sucrose preference in CUMS exposure mouse. Three-time sucrose consumption test was carried out at the end of 0-week, 3-week, and 6-week CUMS exposure. All values given are the mean ± SEM. ^#^ *P* < 0.05 and ^##^ *P* < 0.01 versus control group. ^*^ *P* < 0.05 and ^**^ *P* < 0.01 versus CUMS-vehicle group.

As shown in Figure 2 the value of sucrose consumption was measured three times during the experiment. At the beginning of the experiment (week 0), there were no significant differences among the 6 groups. However, after 3 weeks of CUMS periods, the sucrose consumption of stressed mice was lower than the control group. At the end of CUMS regimen (week 6), sucrose consumption in CUMS group was significantly reduced compared with that in control group. However, the sucrose consumption of CUMS-exposed mice treated for 3 weeks with COR treatment (20 and 40 mg/kg) or FLU (15 mg/kg) increased significantly compared with that in CUMS-vehicle group.

### 3.2. Effects of COR in the Open Field Test

**Figure 3:**
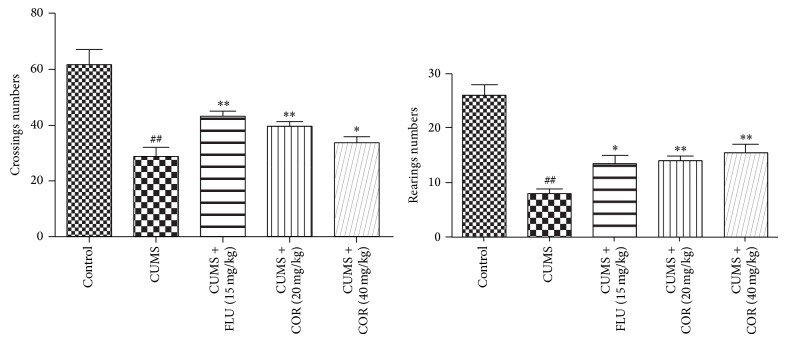
Effect of COR treatment on the number of crossings and rearings in the open field test. All values given are the mean ± SEM. ^#^ *P* < 0.05 and ^##^ *P* < 0.01 versus control group. ^*^ *P* < 0.05 and ^**^ *P* < 0.01 versus CUMS-vehicle group.

Locomotor activity was presented in Figure 3. The results showed that the number of crossings in CUMS-vehicle group mice exhibited a significant reduction versus the control group mice. However, COR (20, 40 mg/kg) or FLU (15 mg/kg) treatments reversed these effects on crossing. CUMS procedure also decreased the number of rearing, and COR or FLU could improve the rearing number.

### 3.3. Effects of COR in the Tail Suspension Test

**Figure 4:**
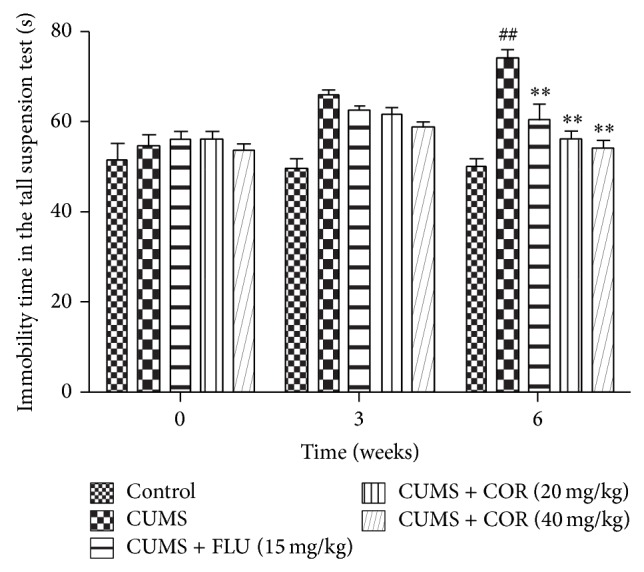
Effects of COR treatment on immobility time in the tail suspension test in mice. Three times sucrose consumption test was carried out at the end of 0-week, 3-week, and 6-week CUMS exposure. All values given are the mean ± SEM. ^#^ *P* < 0.05 and ^##^ *P* < 0.01 versus control group. ^*^ *P* < 0.05 and ^**^ *P* < 0.01 versus CUMS-vehicle group.

Figure 4 showed the effect of COR treatments on the duration of immobility in tail suspension test. At the end of week 0, no significant difference was seen between the 6 groups. However, 5 groups subjected to CUMS appeared to increase immobility time after arriving at the middle of the CUMS procedure (week 3). Finally (week 6), CUMS-vehicle group significantly increased immobility time versus the control group. COR at dose of 20 mg/kg and 40 mg/kg or FLU at dose of 20 mg/kg markedly decreased the immobility time in the tail suspension test compared with the CUMS-vehicle.

### 3.4. Effects of COR in the Forced Swimming Test

**Figure 5:**
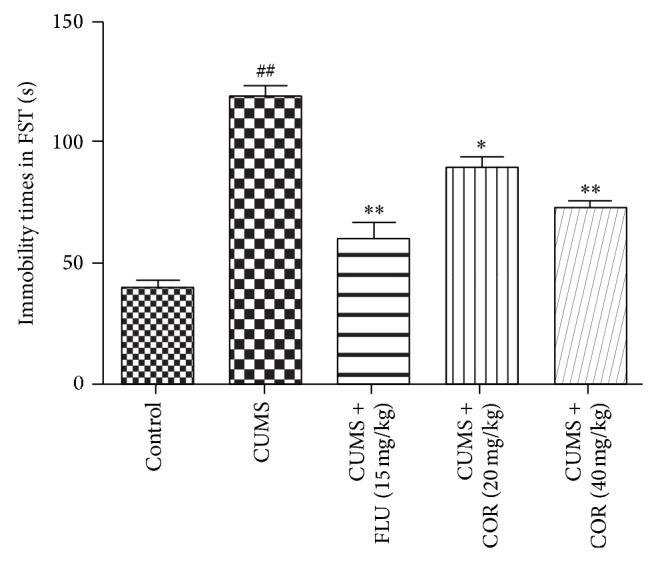
Effect of COR treatment on the immobility times of mice in the forced swimming test. All values given are the mean ± SEM. ^#^ *P* < 0.05 and ^##^ *P* < 0.01 versus control group. ^*^ *P* < 0.05 and ^**^ *P* < 0.01 versus CUMS-vehicle group.

The results of forced swimming test (Figure 5) revealed that CUMS exposure significantly increased in immobility duration compared with control group. COR at dose of 20 mg/kg and 40 mg/kg or the positive control FLU at dose of 20 mg/kg treatments significantly reduced immobility time in the forced swimming test versus CUMS-vehicle group.

### 3.5. Effects of COR on TNF-*α* and IL-6 Levels in the Hippocampus in CUMS Mice

**Figure 6:**
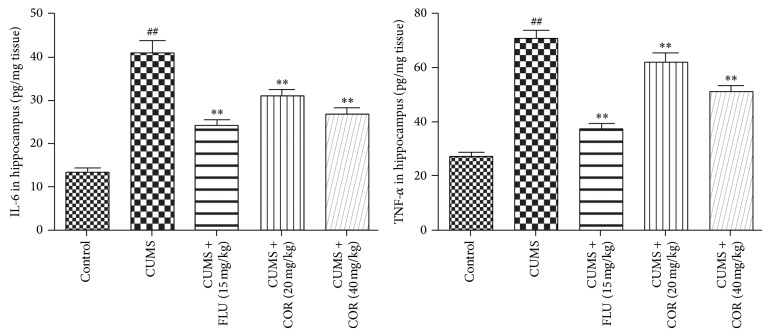
Effects of COR on IL-6 and TNF-*α* levels in the hippocampus in CUMS mice. All values given are the mean ± SEM. ^#^ *P* < 0.05 and ^##^ *P* < 0.01 versus control group. ^*^ *P* < 0.05 and ^**^ *P* < 0.01 versus CUMS-vehicle group.

The effect of COR on TNF-*α* and IL-6 levels in the hippocampus in CUMS mice is shown in Figure 6. CUMS significantly increased TNF-*α* and IL-6 levels concentrations in the hippocampus compared with that in control animals. Administration of COR (20 and 40 mg/kg) was able to reverse the effects of CUMS on TNF-*α* and IL-6 levels concentrations. FLU was able to reverse the effects of CUMS on both DA and 5-HT.

### 3.6. Effects of COR on 5-TH and DA Levels in the Hippocampus in CUMS Mice

**Figure 7:**
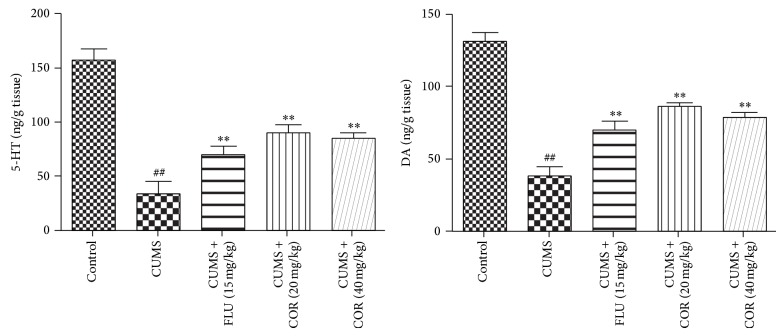
Effects of COR on 5-TH and DA levels in the hippocampus in CUMS mice. All values given are the mean ± SEM. ^#^ *P* < 0.05 and ^##^ *P* < 0.01 versus control group. ^*^ *P* < 0.05 and ^**^ *P* < 0.01 versus CUMS-vehicle group.

The effect of COR on 5-HT and DA levels in the hippocampus in CUMS mice is shown in Figure 7. CUMS significantly reduced 5-HT and DA concentrations in the hippocampus compared with that in control animals. Administration of COR (20 and 40 mg/kg) was able to reverse the effects of CUMS on 5-HT and DA concentrations. FLU was able to reverse the effects of CUMS on both DA and 5-HT.

### 3.7. Effects of COR on NF-*κ*BP65, TNF-*α*, IL-6, 5-HT_2A_R, and BDNF Proteins Expressions in the Hippocampus

**Figure 8:**
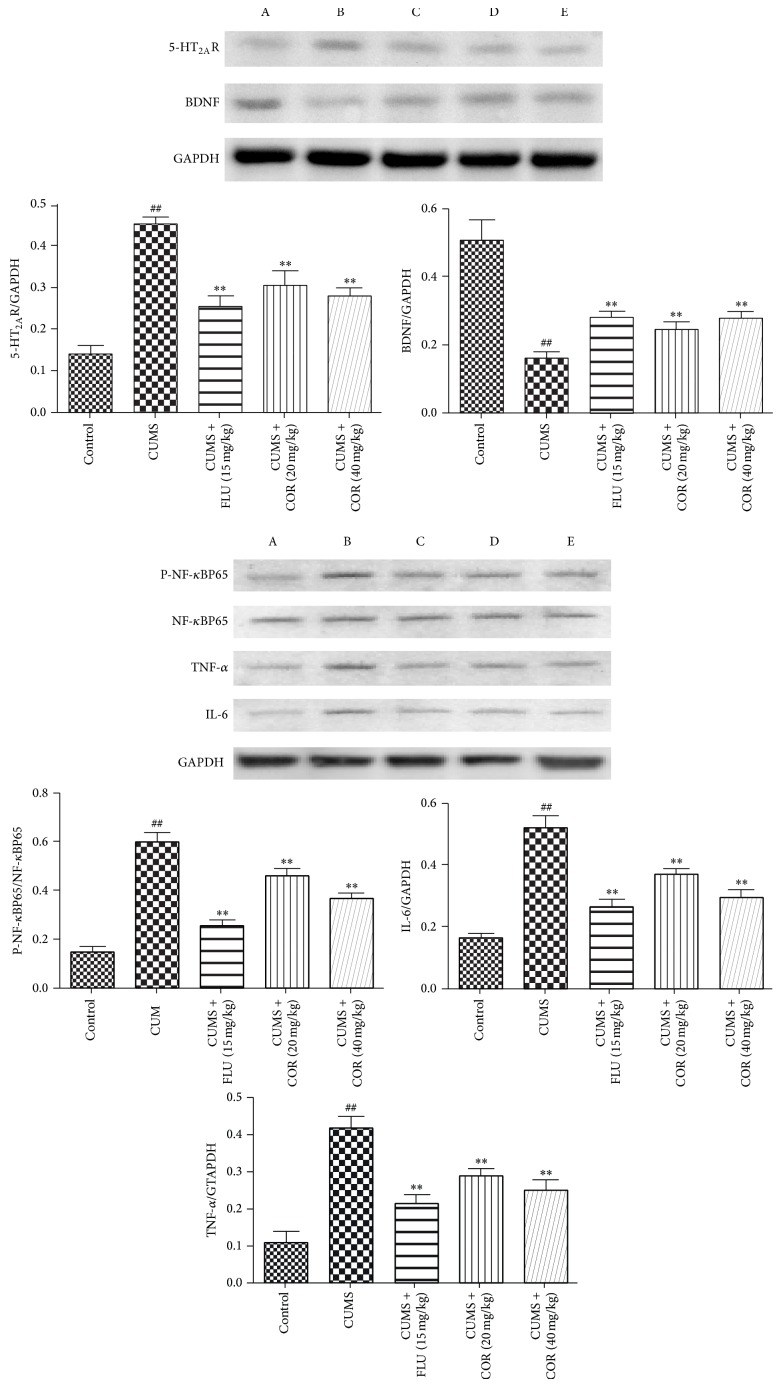
Effects of COR on NF-*κ*BP65, TNF-*α*, IL-6, 5-HT_2A_R, and BDNF proteins expressions in the hippocampus. All values given are the mean ± SEM. ^#^ *P* < 0.05 and ^##^ *P* < 0.01 versus control group. ^*^ *P* < 0.05 and ^**^ *P* < 0.01 versus CUMS-vehicle group. A: Control; B: CUMS; C: CUMS + FLU (15 mg/kg); D: CUMS + COR (20 mg/kg); and E: CUMS + COR (40 mg/kg).

The effect of COR on NF-*κ*BP65, TNF-*α*, IL-6, 5-HT_2A_R, and BDNF proteins levels in the hippocampus in CUMS mice is shown in Figure 8. CUMS significantly increased 5-HT_2A_R, P-NF-*κ*BP65, TNF-*α*, and IL-6 and decreased BDNF in the hippocampus compared with that in control animals; administration of COR (20 and 40 mg/kg) and FLU (15 mg/kg) was able to reverse the effects of CUMS on NF-*κ*BP65, TNF-*α*, IL-6, 5-HT_2A_R, and BDNF.

## 4. Discussion

Currently, we primarily tested the antidepressant-like effects of oral SKA treatments in mice subjected to CUMS. In this work, the CUMS mice model satisfactorily mimicked the depressive status, which was described as the reduction of the sucrose solution consumption and open field activity and increased 5-TH and DA levels, 5-HT_2A_R, and BDNF, respectively. However, COR (20 and 40 mg/kg) administration could oppose the anomalous behavioral changes caused by CUMS process. This study provided new experimental evidence for the antidepressant efficacy of COR in mouse model of CUMS-induced depression.

Chronic stress-induced depression model is a reliable model for studying depression and has been widely utilized in probing the pathological mechanism of depression and screening antidepressant drugs [6]. And CUMS model can mimic the core symptoms of depression, including decreases in sucrose consumption and open field activities and increases in immobility durations (TST and FST) [7].

The sucrose preference test represents the anhedonia-like behavioral change [24]. Anhedonia was modeled by inducing a decrease in responsiveness to rewards reflected by a reduced consumption of sucrose solutions; it is a core symptom of human major depression [25]. In this study, CUMS-exposed mice showed a reduced preference of sucrose solution as compared to control group mice. This result is in agreement with previous findings that mice exposed to CUMS consumed less sucrose solution as compared to control group mice. Treatment with COR significantly reversed this behavioral change, which represents the antidepressant-like effect of COR in CUMS model of depression.

Open field test is widely used to evaluate locomotor and exploratory behaviors in experimental animals [18]; in the open field test, CUMS mice exhibited decreased crossing and rearing which indicated reduced exploration and apathy, respectively, in these animals. COR has a significant ameliorative effect on locomotor behavior in CUMS mice.

The tail suspension test and forced swimming test are behavioral despair tests and had been frequently used to determine depressant-like behavior in rodents after exposure to stress. The immobility time of TST/FST reflects “behavioral despair” as seen in human depression. The data of this investigation showed that mice subjected to chronic stress exhibited a significant prolongation of immobility time in TST/FST. Chronic COR administration significantly decreased the duration of immobility in mice TST/FST, which indicates the antidepressant-like effect.

Several lines of evidence suggest that inflammation plays a role in the pathophysiology of major depression and that anti-inflammatory drugs have antidepressant-like effects [26, 27]. The data of this investigation showed that mice subjected to chronic stress had increased NF-*κ*BP65, TNF-*α*, and IL-6 levels in hippocampus. Chronic COR administration significantly decreased the duration of NF-*κ*BP65, TNF-*α*, and IL-6 in mice, which indicates the antidepressant-like effect.

The 5-HT_2_ receptors in the CNS are thought to be involved in psychiatric disorders such as depression, anxiety, sleep disorders, and hallucinations. The data of this investigation showed that mice subjected to chronic stress had increased 5-HT_2A_R. Chronic COR administration significantly decreased the duration of 5-HT_2A_R in mice, which indicates the antidepressant-like effect.

BDNF, a kind of the nerve growth factor, possesses the ability to support neuronal survival, differentiation, function, and plasticity [28]. Many types of insults induce modifications in brain BDNF expression, and chronic stress application markedly reduces BDNF in hippocampus tissue [29]. CUMS exposure was found to decrease BDNF in the hippocampus of mice, and COR treatments reversed the CUMS-induced decreases in BDNF, which provide a mechanism of action for the antidepressant-like effect of COR.

Our primary findings concluded that COR could improve the depressive-like symptoms induced by CUMS that may be related to regulation of hippocampal 5-HT_2A_R and BDNF proteins expressions. Hence, we consider COR as a potential antidepressant, and its antidepressant activity is partially mediated by the alteration of 5-HT_2A_R and BDNF proteins expressions.
